# Long-term ambient PM_2.5_ exposure associated with cardiovascular risk factors in Chinese less educated population

**DOI:** 10.1186/s12889-021-12163-z

**Published:** 2021-12-10

**Authors:** Jianfeng Lin, Hua Zheng, Peng Xia, Xinqi Cheng, Wei Wu, Yang Li, Chaochao Ma, Guangjin Zhu, Tao Xu, Yali Zheng, Ling Qiu, Limeng Chen

**Affiliations:** 1 Department of Nephrology, State Key Laboratory of Complex Severe and Rare Diseases, Peking Union Medical College Hospital, Chinese Academy of Medical Science and Peking Union Medical College, Beijing, China; 2 Department of Laboratory Medicine, Peking Union Medical College Hospital, Chinese Academy of Medical Science and Peking Union Medical College, Beijing, China; 3 Department of Pathophysiology, Institute of Basic Medical Sciences, Chinese Academy of Medical Sciences and Peking Union Medical College, Beijing, China; 4 Department of Epidemiology and Biostatistics, Institute of Basic Medical Sciences, Chinese Academy of Medical Sciences and Peking Union Medical College, Beijing, China; 5 Department of Nephrology, Affiliated Ningxia People’s Hospital of Ningxia Medical University, Yinchuan, China

**Keywords:** Particulate matter, Diabetes, Hypertension, Hyperlipidemia, Overweight

## Abstract

**Introduction:**

Long-term exposure to ambient air pollution is related to major cardiovascular risk factors including diabetes, hypertension, hyperlipidemia and overweight, but with few studies in high-concentration nations like China so far. We aimed to investigate the association between long-term exposure to ambient fine particulate matter (particles with an aerodynamic diameter ≤ 2.5 μm, PM_2.5_) and major cardiovascular risk factors in China.

**Methods:**

Adult participants with selected biochemical tests were recruited from the Chinese Physiological Constant and Health Condition (CPCHC) survey conducted from 2007 to 2011. Gridded PM_2.5_ data used were derived from satellite-observed data with adjustment of ground-observed data. District-level PM_2.5_ data were generated to estimate the association using multivariate logistic regression model and generalized additive model.

**Results:**

A total of 19,236 participants from the CPCHC survey were included with an average age of 42.8 ± 16.1 years, of which nearly half were male (47.0%). The annual average PM_2.5_ exposure before the CPCHC survey was 33.4 (14.8–53.4) μg/m^3^, ranging from 8.0 μg/m^3^ (Xiwuqi) to 94.7 μg/m^3^ (Chengdu). Elevated PM_2.5_ was associated with increased prevalence of hypertension (odds ratio (OR) =1.022, 95% confidence interval (95%CI): 1.001, 1.043) and decreased prevalence of overweight (OR = 0.926, 95%CI: 0.910, 0.942). Education significantly interacted with PM_2.5_ in association with all the interesting risk factors. Each 10 μg/m^3^ increment of PM_2.5_ was associated with increased prevalence of diabetes (OR = 1.118, 95%CI: 1.037, 1.206), hypertension (OR = 1.101, 95%CI: 1.056, 1.147), overweight (OR = 1.071, 95%CI: 1.030, 1.114) in participants with poor education, but not in well-educated population. PM_2.5_ exposure was negatively associated with hyperlipidemia in all participants (OR = 0.939, 95%CI: 0.921, 0.957). The results were robust in all the sensitivity analyses.

**Conclusion:**

Association between long-term PM_2.5_ exposure and cardiovascular risk factors might be modified by education. PM_2.5_ was associated with a higher prevalence of diabetes, hypertension, and overweight in a less-educated population with time-expose dependency. Long-term exposure to PM_2.5_ might be associated with a lower prevalence of hyperlipidemia.

**Supplementary Information:**

The online version contains supplementary material available at 10.1186/s12889-021-12163-z.

## Introduction

Despite the improvements in the air quality of China, air pollution still plays an important role in public health issues during the past decades. The ambient fine particulate matter (particles with an aerodynamic diameter ≤ 2.5 μm, PM_2.5_) might contribute to 1.1 million (95%CI: 1.0 million to 1.8 million) deaths in China [1], with the most common reason as cardiovascular disease (CVD) mortality [2, 3]. As strong predictors for 10-year risk of CVD [4], the prevalence of hypertension, diabetes, dyslipidemia and overweight was 24.3, 4.3, 49.3 and 32.0%, respectively [5], according to the analysis of 23,010 subjects from Chinese Physiological Constant and Health Condition (CPCHC) conducted from 2007 to 2011. Intervention of risk factors mentioned above would undoubtedly decrease the burden of cardiovascular disease.

Association between the cardiovascular risk factors and PM_2.5_ was vastly investigated, but the result remained inconsistent, with some reporting positive association and some reporting non-association even negative association [6–13]. Moreover, most research was conducted in North America and Europe where annual PM_2.5_ is below 35μg/m^3^, with only a few studies conducted in developing countries like China, which often suffer a much higher concentration and greater domestic variability [14]. Even those conducted in developing countries were region-based [5], hardly covering the full PM_2.5_ spectrum and effects of ethnicity. Meanwhile, previous research suggested that education was a strong modifier of health effects of air pollution [15, 16], and studies exploring the association between PM_2.5_ and cardiovascular risk factors in China mainly focused on population with preliminary or below education attainment [12, 17–20]. Considering evidence for the increasing prevalence of diabetes, hypertension, hyperlipidemia and overweight in those newly industrialized countries were emerging [21–24], it was of great significance to explore the potential associations between PM_2.5_ and these metabolic disorders in a nationwide population-based database.

Hence, we aimed to investigate the association between long-term exposure to PM_2.5_ and the prevalence of major cardiovascular risk factors, including hypertension, diabetes, hyperlipidemia and overweight. Because of the air pollution exposure connected with the social-economic status of the population, the modification effect of education level as an indicator of social-economic status was also analyzed.

## Methods

### Study participants

Participants from the Chinese Physiological Constant and Health Condition (CPCHC) survey were included. Details about CPCHC survey have been reported before [5, 25–27]. Briefly, the CPCHC survey was a cross-sectional, population-based survey covering 82,806 participants from 6 provinces in the north, south, east and west of China (Sichuan, Heilongjiang, Hunan, Inner Mongolia, Yunnan and Ningxia) from 2007 to 2011. Participants were selected under the principle of multistage stratified random cluster sampling, and 35% of them finished the biochemical examination. Adult participants (older than 18 years old) of CPCHC were included. Participants with incomplete information on age, sex, education, ethnicity, smoking status, drinking status, intensity of physical activity, diet types or with missing data on BMI, waist circumference (WC), systolic blood pressure (SBP), diastolic blood pressure (DBP), fasting blood glucose (FBG), serum level of triglyceride, cholesterol, high-density lipoprotein cholesterol (LDL-C), low-density lipoprotein cholesterol (HDL-C) were excluded. Before data collection, we obtained the written informed consent from each participant. The protocol was in line with the Helsinki Declaration and approved by the ethic review board of the Institute of Basic Medical Sciences, Chinese Academy of Medical Sciences.

### Data collection and definitions

We designed a standard questionnaire to gather the necessary information, including age, sex, education, ethnicity, smoking status, drinking status, intensity of physical activity and diet types. A participant was categorized as less-educated if one only received preliminary or below education or no schooling. The minority is composed of ethnic groups other than Han ethnicity. Current smoker and current drinker were defined as participants with self-reported responses of “yes” to the question “Do you smoke cigarettes now?” and “Do you drink now?” respectively. A lower threshold of cigarettes smoked was one, and the minimal drinking amount for liquor was 150 g or less, 1 bottle or less for beer, and 50 g or less for other alcoholic beverages. Intensity of physical activity was defined as low, moderate, or high according to the self-reported labor or sports intensity on average. Diet types include sugar-rich diet, salt-rich diet, spicy diet, fat-rich diet, light diet, and each was defined as self-reported “yes” to the questions “Are your daily diet sugar-rich?”, “Are your daily diet salt-rich?”, “Are your daily diet spicy?”, “Are your daily diet fat-rich?”, “Are your daily diet light?”, respectively. Residence (urban, rural) and region (south, north) were determined by the surveyed location.

Meanwhile, physical examination included measurement of height, weight, BMI, WC, SBP and DBP. BMI was defined as weight (kg) divided by square of height (m^2^). WC was measured in a standing position with soft inelastic tape at the end of a gentle expiration. We asked each participant to rest quietly for at least 10 min before blood pressure measurement by trained medical personnel using an electric sphygmomanometer (OMRON, HEM-7000). The documented blood pressure was average value of the three-time test. We asked each participant to fast overnight before the biochemical test. The cholesterol, HDL-C, LDL-C, and triglycerides were measured by Beckman AU Series Automatic Biochemical Analyzer (Japan) and Sekisui Medical (Japan) reagents, while FBG by Beckman AU reagents.

Diabetes was diagnosed as FBG ≥7.0 mmol/L, or with current anti-diabetes drug use. Hypertension was diagnosed as an average SBP ≥ 140 mmHg and/or DBP ≥ 90 mmHg, or with anti-hypertensive drug use. Hyperlipidemia was diagnosed as cholesterol ≥5.2 mmol/L. Overweight was diagnosed as BMI ≥ 24 kg/m^2^.

A directed acyclic graph (DAG, Fig. S1) was used to identify potential confounding variables using DAGitty v1.0 software. The covariates included in data analysis were as followed: age (year), sex, education (preliminary school or below, middle school, high school, college or above), ethnicity (Han, Yi, Hui, Mongolian, Tibetan, Korean, Tujia, Miao, others), smoking status (current smoker, nonsmoker), drinking status (current drinker, nondrinker), intensity of physical activity (low, moderate, high), diet types (sugar-rich diet, salt-rich diet, spicy diet, fat-rich diet, light diet), residence (urban, rural), region (south, north).

### Air pollution exposure measurements

We obtained PM_2.5_ data from the website of Atmospheric Composition Analysis Group (http://fizz.phys.dal.ca/~atmos/martin/?page_id=140). The detailed process of PM_2.5_ data generation was the same as reported [28, 29]. The gridded PM_2.5_ data recording annual average PM_2.5_ concentration was transformed from GEOSChem model and satellite observations of aerosol optical depth (AOD), with a resolution of 0.1° × 0.1° (latitude times longitude). A PM_2.5_ dataset ranging from 2000 to 2016 was then corrected by the raw data with ground-based PM_2.5_ data in China from May 2014 to December 2016, using the geographically weighted regression (GWR) method. The gridded PM_2.5_ data from 2001 to 2010 in China used in this study were then processed using ArcMap (version 10.6.1, ESRI Inc). District-level annual average PM_2.5_ data 1 year, 2 years, 3 years, 4 years and 5 years before the CPCHC survey was generated to represent the exposure level for residents in each surveyed city.

### Statistical analysis

Continuous variables with normal distribution were presented as the mean (standard deviation) and compared by independent sample t-test. Skewed distributed variables were presented as the median (interquartile range) and analyzed by the Mann-Whitney U test. Categorical data were presented as percentages and compared by the χ^2^ test.

Logistic regression analyses were used to estimate the association between 5-year PM_2.5_ (each 10 μg/m^3^ increase) and major cardiovascular risk factors, including diabetes, hyperlipidemia, hypertension and overweight. In crude model, no covariates were included. In multivariate logistic regression model, age, sex, education, ethnicity, smoking status, drinking status, intensity of physical activity and diet types were included as covariates. Interaction was tested by introducing interaction terms into the overall model using likelihood ratio test. Generalized additive model was used to explore the exposure-response relationship between PM_2.5_ and major cardiovascular risk factors. A series of sensitivity analyses were conducted. First, we constructed regression models adjusted by introducing covariates stepwise. Second, we used PM_2.5_ exposure defined from 1-year to 5-year exposure value as well as dichotomous PM_2.5_ exposure to assess the association between PM_2.5_ and cardiovascular risk factors. We dichotomized PM_2.5_ exposure with a cutoff value at 35 μg/m^3^ because an annual mean PM_2.5_ concentration of 35 μg/m^3^ was the interim targets level by WHO Air quality guidelines [30]. Third, stratification analyses according to disease status (yes or no), residence or region were conducted and a cross-product term were also added into the overall model to test for interaction. The *P*-values were two-sided, and *P* < 0.05 were considered statistically significant. All statistical analyses were performed with R (Version 4.0.3, R Core Team).

## Results

**Fig. 1:**
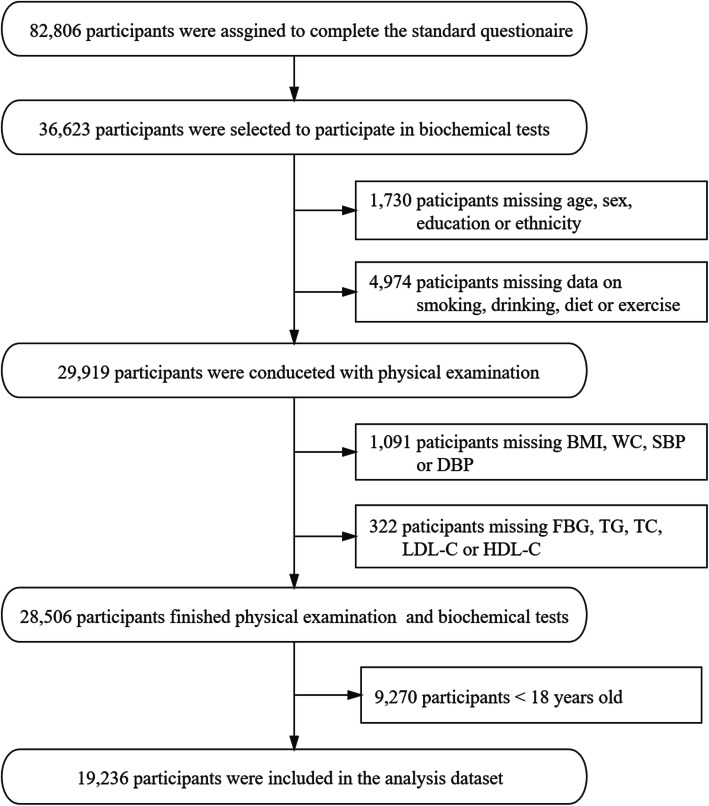
Flow diagram of participant inclusion

82,806 participants were in enrolled in the CPCHC survey (Fig. 1). Among 36,623 participants who were selected to participate in biochemical tests, 29,919 of them completed a standard questionnaire surveying demographic information (age, sex, education, ethnicity, etc.) and lifestyles (smoking status, drinking status, intensity of physical activity, diet types, etc.). Excluding 1091 participants with missing data on BMI, WC, SBP or DBP and 322 participants without data on FBG, TG, TC, LDL-C or HDL-C, the remaining 19,236 participants whose age older than 18 years old were selected for the present study.

The mean age was 42.8 ± 16.1 years (Table 1). 53.0% of the studied population were female. 22.2% of the participants attained preliminary school or below education, and 58.6% of the studied population attained at least a high school education. Most of the participants were Han ethnicity (62.9%), nonsmoker (78.9%), nondrinker (76.9%), and moderate physical activity (53.8%). A small proportion of the participants reported diet types as spicy diet (22.0%), light diet (18.7%), sugar-rich diet (13.0%), salt-rich diet (10.7%), or fat-rich diet (9.0%). Nearly half of the participants were urban residents (50.8%) and came from southern China (53.2%). In general, 755 (3.9%), 4383 (22.8%), 5391 (28.0%) and 7727 (40.2%) of the participants had diabetes, hypertension, hyperlipidemia, and overweight, respectively. The median concentration of PM_2.5_ was 33.4 (IQR 14.8–53.4) μg/m^3^ in the surveyed cities, covering northern, southern, eastern and western China (Fig. S2). Chengdu was reported with the highest concentration (94.7 μg/m^3^), while the lowest level of PM_2.5_ exposure was documented in Xiwuqi (8.0 μg/m^3^) (Fig. S3). The prevalence of hypertension and hyperlipidemia were significantly lower in the urban region (Table S1). The prevalence of hypertension and overweight were significantly lower in southern China, but the prevalence of hyperlipidemia was higher (Table S2).

**Table 1:** Demographic and health characteristics of participants with different exposure of PM_2.5_, China, 2006–2010

	Overall population	PM_**2.5**_ < 35 μg/m^**3**^	PM_**2.5**_ ≥ 35 μg/m^**3**^	*P*
N, *n* (%)^*a*^	19,236 (100)	10,010 (52.0)	9226 (48.0)	
Age, mean ± SD^*b*^	42.8 ± 16.1	41.8 ± 15.1	43.9 ± 17.1	< 0.001
Age ≥ 65 years old, *n* (%)^*a*^	2102 (10.9)	848 (8.5)	1254 (13.6)	< 0.001
Sex (Male), *n* (%)^*a*^	9041 (47.0)	4530 (45.3)	4511 (48.9)	< 0.001
Education, *n* (%)^*a*^				< 0.001
Preliminary school or below	4263 (22.2)	3098 (31.0)	1165 (12.6)	
Middle school	3709 (19.3)	2032 (20.3)	1677 (18.2)	
High School	4245 (22.1)	1975 (19.7)	2270 (24.6)	
College or above	7019 (36.5)	2905 (29.0)	4114 (44.6)	
Ethnicity, *n* (%)^*a*^				< 0.001
Han	12,108 (62.9)	5419 (54.1)	6689 (72.5)	
Yi	1981 (10.3)	1972 (19.7)	9 (0.1)	
Hui	1492 (7.8)	139 (1.4)	1353 (14.7)	
Mongolian	1065 (5.5)	1051 (10.5)	14 (0.2)	
Tibetan	664 (3.5)	657 (6.6)	7 (0.1)	
Korean	643 (3.3)	554 (5.5)	89 (1.0)	
Tujia	616 (3.2)	5 (0.0)	611 (6.6)	
Miao	377 (2.0)	11 (0.1)	366 (4.0)	
Others	290 (1.5)	202 (2.0)	88 (1.0)	
Current smoker, *n* (%)^*a*^	4064 (21.1)	2243 (22.4)	1821 (19.7)	< 0.001
Current drinker, *n* (%)^*a*^	4443 (23.1)	2399 (24.0)	2044 (22.2)	0.003
Physical activity, *n* (%)^*a*^				< 0.001
Low	2679 (13.9)	1322 (13.2)	1357 (14.7)	
Moderate	10,350 (53.8)	5230 (52.2)	5120 (55.5)	
High	6207 (32.3)	3458 (34.5)	2749 (29.8)	
Sugar-rich diet, *n* (%)^*a*^	2498 (13.0)	1411 (14.1)	1087 (11.8)	< 0.001
Salt-rich diet, *n* (%)^*a*^	2059 (10.7)	1100 (11.0)	959 (10.4)	0.183
Spicy diet, *n* (%)^*a*^	4226 (22.0)	2073 (20.7)	2153 (23.3)	< 0.001
Fat-rich diet, *n* (%)^*a*^	1735 (9.0)	987 (9.9)	748 (8.1)	< 0.001
Light diet, *n* (%)^*a*^	3594 (18.7)	1616 (16.1)	1978 (21.4)	< 0.001
Residence (urban), *n* (%)^*a*^	9777 (50.8)	4369 (43.6)	5408 (58.6)	< 0.001
Region (south), *n* (%)^*a*^	10,224 (53.2)	4942 (49.4)	5282 (57.3)	< 0.001
PM_2.5_, median (IQR), μg/m^3 *c*^	33.4 (14.8–53.4)	14.8 (13.8–21.8)	53.4 (48.9–62.8)	< 0.001
Diabetes, *n* (%)^*a*^	755 (3.9)	329 (3.3)	426 (4.6)	< 0.001
Hypertension, *n* (%)^*a*^	4383 (22.8)	2204 (22.0)	2179 (23.6)	0.008
Hyperlipidemia, *n* (%)^*a*^	5391 (28.0)	3193 (31.9)	2198 (23.8)	< 0.001
Overweight, *n* (%)^*a*^	7727 (40.2)	4030 (40.3)	3697 (40.1)	0.790

Abbreviation: IQR, interquartile range; SD, standard deviation

^*a*^ Compared by the χ2 test

^*b*^ Compared by independent sample t-test

^*c*^ Compared by the Mann-Whitney U test

**Fig. 2:**
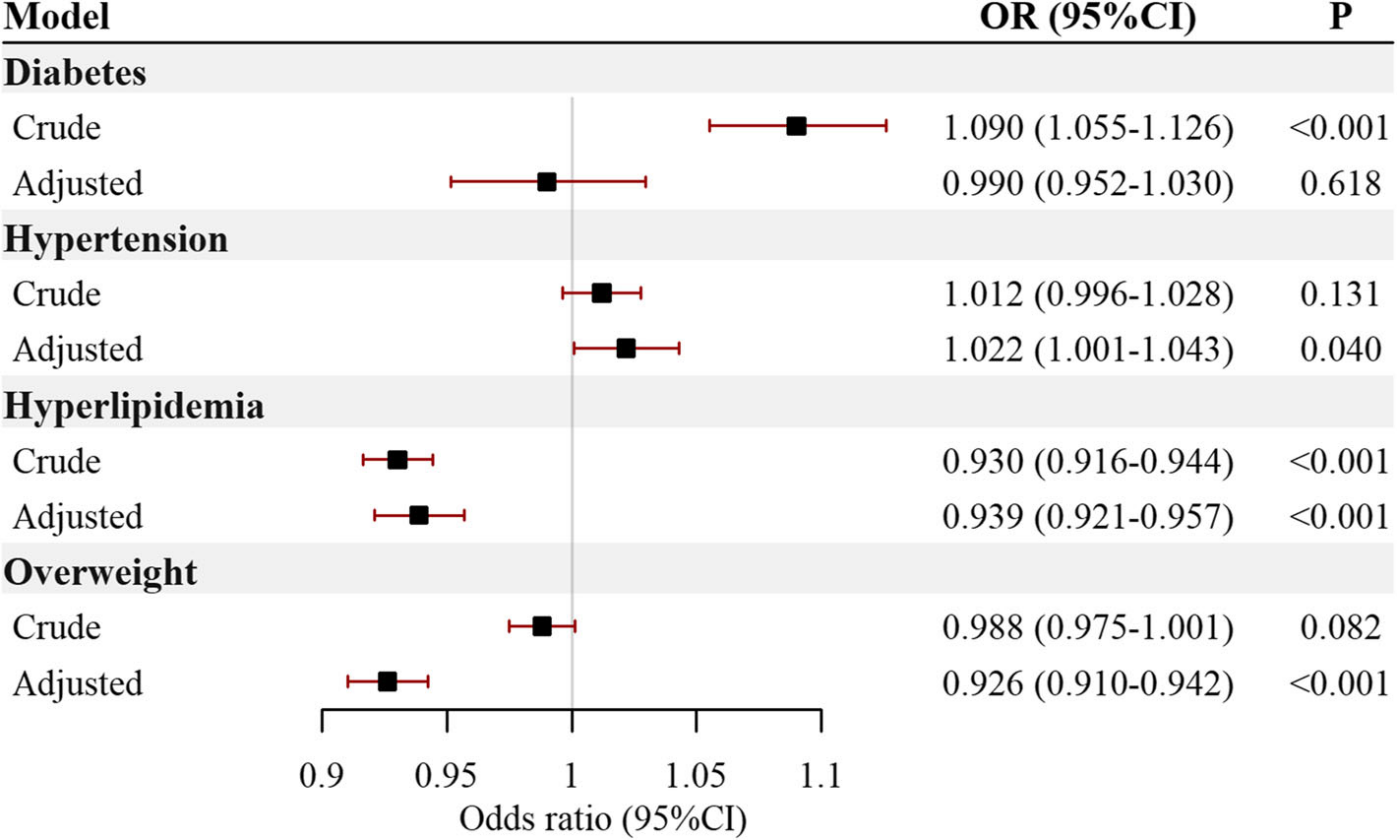
Association between PM_2.5_ exposure and cardiovascular risk factors prevalence. The odds ratios and relevant 95% CI were scaled to each 10 μg/m^3^ PM_2.5_ exposure and calculated by multivariable logistic regression in crude model, and further adjusted for age, sex, education, ethnicity, smoking status, drinking status, intensity of physical activity, and diet types in adjusted model

**Fig. 3:**
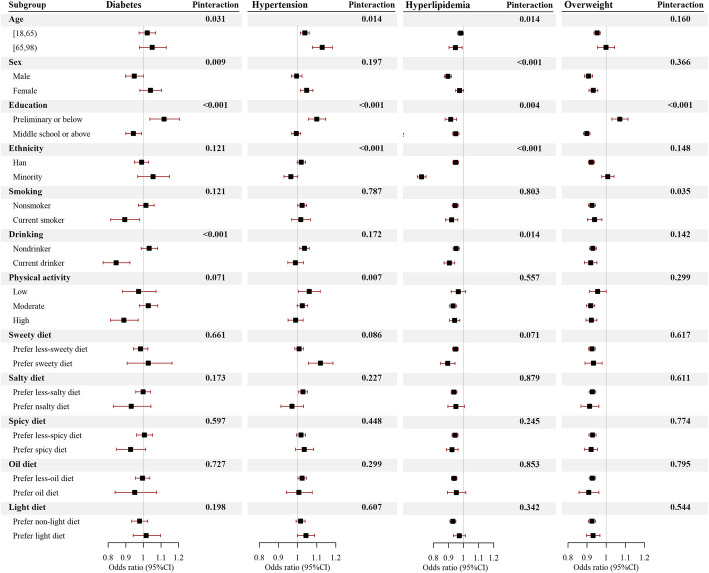
Association between PM_2.5_ exposure and cardiovascular risk factors prevalence stratified by covariates. Population was stratified by age, sex, education, ethnicity, smoking status, drinking status, intensity of physical activity, and diet types. The odds ratios and relevant 95% CI were scaled to each 10 μg/m^3^ PM_2.5_ exposure and calculated by multivariable logistic regression, and further adjusted for age, sex, education, ethnicity, smoking status, drinking status, intensity of physical activity, and diet types. The significance of interaction effect was tested by introducing an interaction term in the regression model

**Fig. 4:**
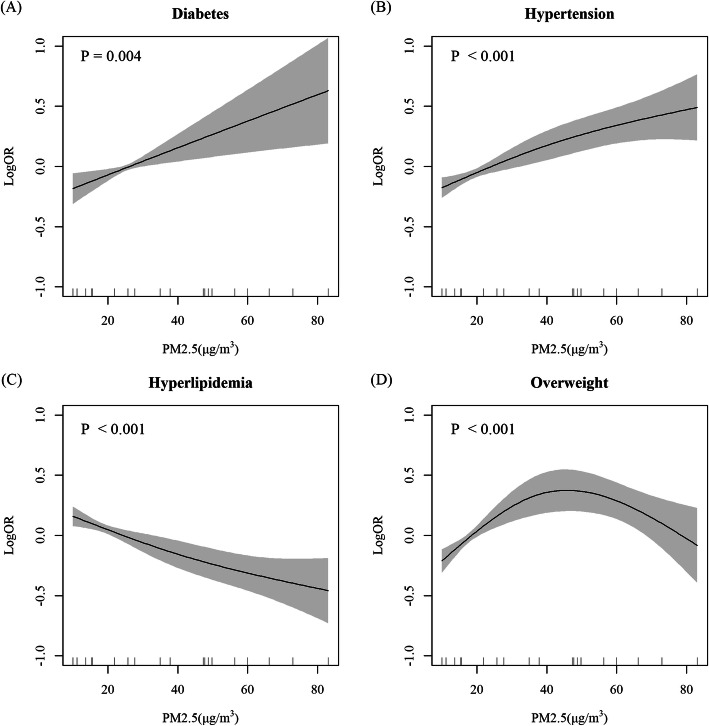
Exposure-response relationship between PM_2.5_ exposure and cardiovascular risk factors prevalence in population with preliminary or below education. (A) diabetes; (B) hypertension; (C) hyperlipidemia; (D) being overweight. The exposure-response relationship was calculated by generalized additive model, and further adjusted by age, sex, ethnicity, smoking status, drinking status, intensity of physical activity, diet types. Knots used in the generalized additive model was 3. *P* value was denoted in each panel

In general, each 10 μg/m^3^ increment of 5-year PM_2.5_ exposure was associated with hypertension (OR = 1.022, 95%CI: 1.001, 1.043), hyperlipidemia (OR = 0.939, 95%CI: 0.921, 0.957), and overweight (OR = 0.926, 95%CI: 0.910, 0.942), after adjustment of age, sex, education, ethnicity, smoking, drinking, intensity of physical activity and diet types **(**Fig. 2**)**. Education was the only covariate that significantly interacted with PM_2.5_ on association with all the interesting risk factors (P for interaction: diabetes< 0.001, hypertension < 0.001, hyperlipidemia = 0.004, overweight < 0.001, respectively.) **(**Fig. 3**)**. In general, PM_2.5_ was associated with increased prevalence of diabetes (OR = 1.118, 95%CI: 1.037, 1.206), hypertension (OR = 1.101, 95%CI: 1.056, 1.147) and overweight (OR = 1.071, 95%CI: 1.030, 1.114) in population with preliminary or below education attainment, but decreased prevalence of hyperlipidemia (OR = 0.915, 95%CI: 0.878, 0.953). The exposure-response analysis revealed that PM_2.5_ had near-linear relationship with diabetes, hypertension and hyperlipidemia in less-educated population, but the prevalence of overweight was firstly increased and then decreased with the increase of PM_2.5_ exposure **(**Fig. 4**)**. The corresponding relationship in the general population, as well as in the well-educated population, was different from the less-educated, especially the association between PM_2.5_ and diabetes in the less-educated, the general, and the well-educated population, which were positive, null and inverse respectively (Figs. S4, S5).

**Fig. 5:**
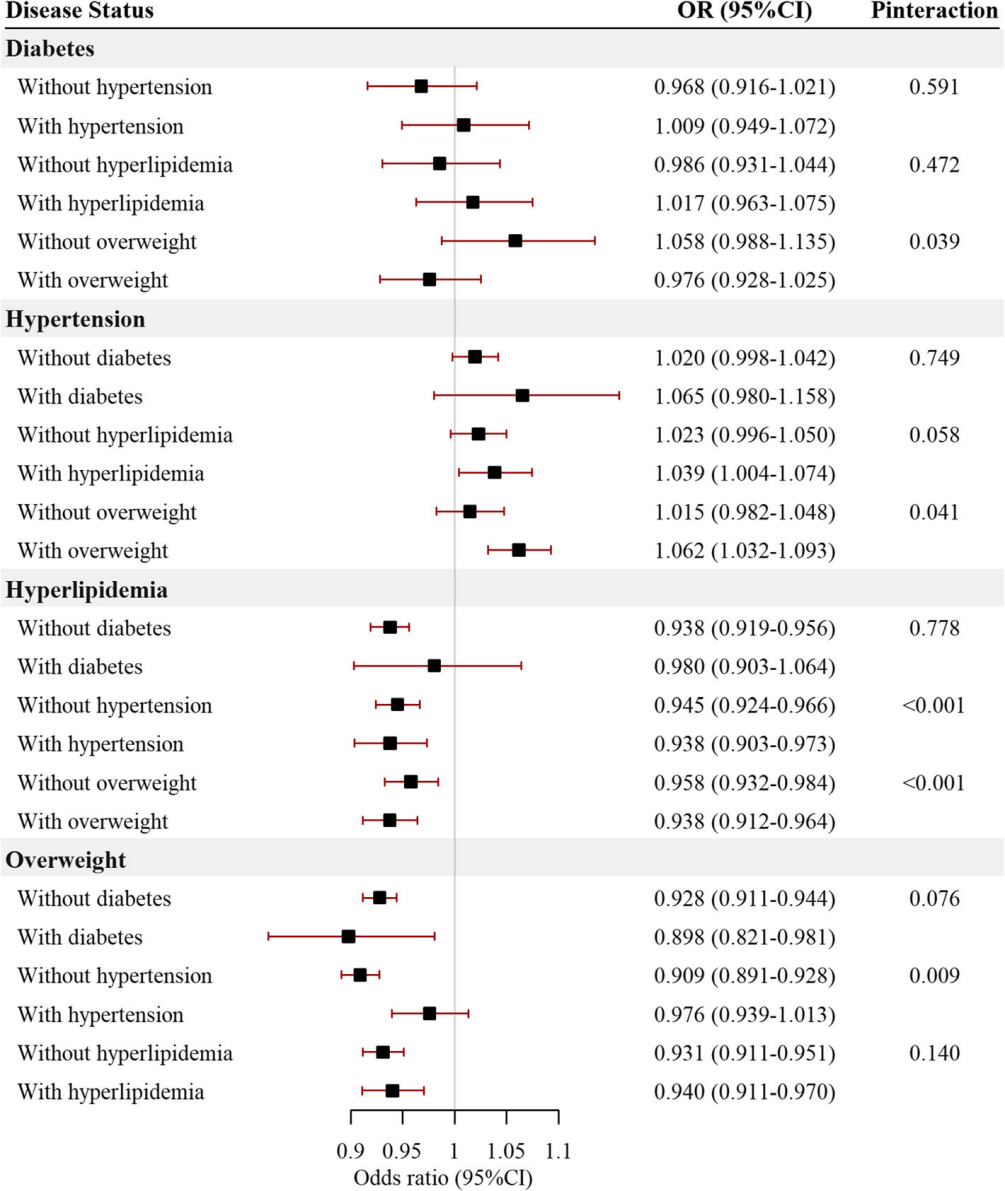
Association between PM_2.5_ exposure and cardiovascular risk factors prevalence stratified by cardiovascular risk factors. The odds ratios and relevant 95% CI were scaled to each 10 μg/m^3^ PM_2.5_ exposure and calculated by multivariable logistic regression, and further adjusted for age, sex, education, ethnicity, smoking status, drinking status, intensity of physical activity, and diet types

In sensitivity analyses, we introduced the covariates stepwise in the regression models, and the regression result tended to be stable after being adjusted by age, sex, education and ethnicity, and model 7 was used in this study which included age, sex, education, ethnicity, smoking status, drinking status, intensity of physical activity and diet types (Fig. S6). The PM_2.5_ exposure values defined by different time duration were highly correlated, and the model stayed stable after changing the PM_2.5_ exposure used in the regression model (Fig. S7). Meanwhile, the association between binary PM_2.5_ exposure and cardiovascular risk factors was similar to the results of continuous PM_2.5_ exposure (Fig. S8). Stratified by disease status, we found that the general trend of PM_2.5_ association with each risk factor was generally stable. However, hypertension (P for interaction < 0.001 for hyperlipidemia; P for interaction = 0.009 for overweight) and overweight (P for interaction = 0.039 for diabetes; P for interaction = 0.041 for hypertension; P for interaction < 0.001 for hyperlipidemia) were the risk factors that interacted with PM_2.5_ **(**Fig. 5**)**. Residence strongly interacted with PM_2.5_ on all the risk factors (Fig. S9), but region significantly interacted with PM_2.5_ only on hypertension (Fig. S10). Besides, in models adjusted for age, sex, education, ethnicity, smoking, drinking, the intensity of physical activity, diet types, and residence, the results remained robust compared to models without residence adjustment (Figs. S11, S12, S13, S14).

## Discussion

In this study, we firstly used a multi-provincial and cross-ethnical study in China to demonstrate that long-term high concentration PM_2.5_ exposure (8.0–94.7 μg/m^3^) was positively associated with diabetes, hypertension, and overweight in the participants with poor education, but not in the well-educated population of Chinese adults. After adjusting for several potential confounders, the negative association between PM_2.5_ exposure and hyperlipidemia remained robust and significant among 19,236 multiethnic Chinese adults.

Our findings suggest that higher long-term PM_2.5_ exposure was positively associated with diabetes, hypertension, and overweight in the participants with poor education. Significant positive association between PM_2.5_ and diabetes, hypertension and overweight was also reported in several cohort studies, including a few Chinese cohorts [7, 9, 31–33], and regions with lower PM_2.5_ concentration, like American [34, 35], Canadian [36, 37], and Korean [38]. Our results were also consistent with several cross-sectional studies among the Chinese population [12, 17–20]. Participants with preliminary or below education attainment accounted for 63.23, 63.5, 59.5, 69.2 and 68.1% on Yang’s study on diabetes [20], Liu’s study on diabetes [19], Lin’s study on hypertension [17], Liu’s study hypertension [18], and Yang’s study on overweight [12].

However, we did not observe significant association between PM_2.5_ and diabetes or hypertension in participants with middle school or above education attainment. This discrepancy may partly be due to education was a strong modifier of the health effects of air pollution [15]. As an important indicator of socio-economic status (SES), education may modify the health effects of air pollution in several ways. First, the less-educated were more associated with higher pollution exposure in their working environment, such as mining, heavy industrial, construction and other outdoor jobs. Second, smoke produced by traditional biofuel burning and insufficient ventilation in kitchen may aggravate household pollution, which is also more common in the low SES population. Evidence also exists suggesting that individuals with lower education are generally more exposed to more PM_2.5_ pollution than those with higher education in the workday, or the workplace, even at home [39]. Third, lower education groups might lack access to medical care and awareness of disease, resulting in worse health outcomes [40]. Fourth, lower SES was related to higher stress levels, thus affecting the neuroendocrine dysfunction and leading to development of diabetes and hypertension [16, 41]. Several other cohort studies also investigated the association between diabetes and hypertension but made null conclusions, including American cohorts [6, 8], and German cohort [42], which may partly be due to modification of education attainment. We also reported that PM_2.5_ would decrease the prevalence of overweight in better-educated groups, which was consistent with a previous study revealing populations with high SES were less likely to be obese and vice versa [43]. Income-level and peer effects can partly explain the modification of education on obesity [44]. Dose-response curve revealed a non-linear effect of PM_2.5_ on overweight, which was also reported in previous literature. According to de Bont et al. [45], the association between PM_2.5_ and obesity was most evident for PM_2.5_ from 20 to 28 μg/m^3^ but not higher, a range lower than the median value than our research. This could be attributed to both the dosage saturation effect and other social factors. In the past few decades, air pollution, especially PM_2.5_ has drawn extensive attention in China due to information spread by social media, television, newspaper, etc. Corresponding initiatives against air pollution might thus result in better health conditions, especially those who perceived earlier and took it carefully, which coincided with the high-educated population in this study. Nonetheless, such a protective effect might be absent in lower PM2.5 exposure region, which might explain the non-linear effect. Similar effect could be observed for hypertension, which also showed a protective trend at the high end of the PM_2.5_ spectrum. However, caution should be taken as this subgroup has smaller sample size. More investigation should be taken before any definite conclusion.

We also observed that health effect of PM_2.5_ was different between the male and the female. The difference between males and female was explored in previous studies [15, 46, 47], but the patterns remained inconsistent. Region interacted with PM_2.5_ on the prevalence of hypertension, which might be explained by the association between region and temperature, while the lower outdoor temperature is strongly associated with a higher prevalence of hypertension [48, 49]. Urban-rural disparities in the prevalence of diabetes [50], hypertension [51], dyslipidemia [52] and overweight [53] in China have been reported. Urban or rural residence strongly interacted with PM_2.5_ on the prevalence of each cardiovascular risk factor. Residence was also an indicator of SES as education, and educational attainment and residence were also highly correlated in this study (data not shown). The rural-to-urban migration trend [54] and urbanization degree would change the risk of acquiring chronic diseases [55], but relevant data were not recorded in CPCHC survey, which may result in biased estimation of rural-urban effect. These results may have occurred by chance or may reveal underlying challenges in analyses of air pollution where multiple, highly correlated attributes of air pollutions of an individual’s environment can potentially play a role.

Only a few studies investigated the relationship between long-term PM_2.5_ exposure and cholesterol or hyperlipidemia, and the findings remained inconsistent [10, 11, 13, 56–58]. In this study, we demonstrated that PM_2.5_ exposure was associated with decrease in the prevalence of hyperlipidemia in both low and high educational groups. However, no significant association was reported in a cross-sectional study in the USA [58], and a 12-month PM_2.5_ exposure cohort study of midlife American women [10]. Meanwhile, previous studies conducted in different provinces of China revealed a significant positive association between PM_2.5_ exposure and cholesterol level [11, 56, 57]. The inconsistency among those studies may result from the following reasons. Firstly, annual PM_2.5_ was below 35μg/m^3^ in most research mentioned above, much lower than our research. Even previous studies conducted in China were province-based and had not covered the full spectrum of PM_2.5_ concentrations considered in our analyses. This may suggest the association between PM_2.5_ and lipid might be concentration-dependent. Secondly, sex may influence the health effect of PM_2.5_, and our analysis for female participants revealing null association as in the midlife-American women study [10]. Thirdly, ethnicity may be a strong mediator of metabolic syndrome [59], but previous Chinese studies included only participants in eastern China, including Taiwan [56], Liaoning [57], and Henan [11], and the majority of participants were Han ethnicity. Health-related behaviors and lifestyle factors of different ethnicity would shape different lipid profiles, thus modifying the effect of air pollution [60]. Mao et al. also reported that increased PM_2.5_ pollution was associated with decreased TG in the rural population of China [11], which also indicated the difficulty to explore the health effect of PM_2.5_.

The mechanisms underlying PM_2.5_ and cardiovascular risk factors have been vastly studied. Inhalation of particulate matter by lung can lead to autonomous nervous system imbalance, lung inflammation and alveolocapillary translocation. It was also followed by releasing of catecholamines and systemic inflammation, resulting in various subclinical and clinical disorders, including diabetes and hypertension, as indicated in our study focusing on participants with preliminary or below school education [61]. Recent experimental evidence suggested that long-term and heavy PM_2.5_ exposure would reduce total cholesterol level and triglyceride level, leading to lipid peroxidation and dyslipidemia, which may partly explain the discrepancy between our study and previous conclusions [62]. Another recent study on mice revealed that female mice were more susceptible to ambient PM_2.5_ exposure-induced hepatic cholesterol accumulation, indicating that health effect of PM_2.5_ may be sex-dependent [63]. The mechanisms underlying the association between PM_2.5_ and cholesterol and body weight should be furthered explored.

To our best knowledge, this is the largest multi-provincial and multi-ethnic study in developing countries to date and covered the broadest PM_2.5_ spectrum, providing evidence for health effect of PM_2.5_ in both high concentration and low concentration. Previous studies conducted in American and European countries fail to cover the impact of high PM_2.5_ concentration (>35μg/m^3^) on CVD risk factors. This research also demonstrated population with lower education might be especially susceptible to PM_2.5_ association with CVD risk factors.

### Limitations

We acknowledge that our study has several limitations. Firstly, designed as a cross-sectional study, we cannot establish a cause-effect relationship between PM_2.5_ and cardiovascular risk factors. Secondly, we only evaluated the effect of PM_2.5_ based on data at district-level but not at individual-level. The association of other air pollutants should be explored and more precise measurement of personal exposure method should be developed. Thirdly, we only tested the fast glucose once and combined with the medical history of diabetes, did not measure the glycosylated hemoglobin and postprandial blood glucose, which might misestimate the prevalence of diabetes. Meanwhile, covariates including current smoking status, current drinking status, intensity of physical activity and diet types were self-reported and misclassification bias was inevitable, which may influence the association between PM_2.5_ and cardiovascular risk factors. Fourthly, although many potentials confounders were taken into consideration, we did not acquire adequate socioeconomic factors other than education and comorbid diseases, which may modify the exposure-outcome associations. Fifthly, the effects of other air pollutants, such as PM_1_, PM_10_, and NO_2_, are also deserved exploration. Due to lack of accessibility to other air pollutants measurement, health effects of PM_1_, PM_10_, and NO_2_, were not analyzed in this study.

## Conclusion

Long-term PM_2.5_ exposure was associated with higher prevalence of diabetes, hypertension, and overweight in the less-educated general population, which contributed to the cardiovascular disease. The robust protection effect of high-concentration PM_2.5_ exposure in hyperlipidemia needs more studies.

## Supplementary Information

**Additional file 1.**

## Data Availability

The datasets analyzed during the current study are available from the corresponding author on reasonable request.
