# Supplementary material for: Long-term ambient PM2.5 exposure associated with cardiovascular risk factors in Chinese less educated population
Source: BMC Public Health. 2021 Dec 10;21:2241. doi: 10.1186/s12889-021-12163-z (PMC8662859; doi:10.1186/s12889-021-12163-z)
Supplement: Supplementary file 1 — Additional file 1. [file 12889_2021_12163_MOESM1_ESM.docx]

# Legend

**Supplemental Material**

**Table S1 Prevalence of cardiovascular risk factors in urban and rural residents**

**Table S2 Prevalence of cardiovascular risk factors in north and south region**

**Figure S1 Proposed Directed acyclic graph for the association between PM2.5 and CVR (cardiovascular risk factors)**

**Figure S2 Annual average PM_2.5_ concentration in 2008, China and distribution of surveyed cities.**

**Figure S3 Distributions of PM_2.5_ concentration in the surveyed cities (2001-2010).**

Boxes covered the 25–75th percentile, with a centerline for the median concentration. Whiskers extend to the highest observation within 3 IQRs (interquartile ranges) of each box. The extreme data were shown as dots.

**Figure S4 Exposure-response relationship between PM_2.5_ exposure and cardiovascular risk factors prevalence in general population.**

(A) diabetes; (B) hypertension；(C) hyperlipidemia; (D) overweight. The exposure-response relationship was calculated by generalized additive model, and further adjusted by age, sex, education, ethnicity, smoking status, drinking status, intensity of physical activity, diet types. Knots used in the generalized additive model was 3. P value was denoted in each panel.

**Figure S5 Exposure-response relationship between PM_2.5_ exposure and cardiovascular risk factors prevalence in population with middle or above education.**

(A) diabetes; (B) hypertension; (C) hyperlipidemia; (D) being overweight. The exposure-response relationship was calculated by generalized additive model, and further adjusted by age, sex, ethnicity, smoking status, drinking status, intensity of physical activity, diet types. Knots used in the generalized additive model was 3. P value was denoted in each panel.

**Figure S6 Association between PM_2.5_ exposure and cardiovascular risk factors prevalence determined by different logistic regression models.**

The odds ratios and relevant 95% CI were scaled to each 10μg/m^3^ PM_2.5_ exposure and calculated by univariate or multivariable logistic regression.

Model 1 adjusted for: none

Model 2 adjusted for: age, sex

Model 3 adjusted for: Model 2 + education, ethnicity

Model 4 adjusted for: Model 3 + smoking status, drinking status

Model 5 adjusted for: Model 3 + intensity of physical activity

Model 6 adjusted for: Model 3 + diet types

Model 7 adjusted for: Model 3 + smoking status, drinking status, intensity of physical activity, diet types

Model 8 adjusted for: Model 7 + all other diseases

Model 9 adjusted for: Model 3 + residence (rural/ urban)

Model 10 adjusted for: Model 3 + region (south/ north)

Model 11 adjusted for: Model 7 + residence (rural/ urban)

Model 12 adjusted for: Model 7 + region (south/ north)

**Figure S7 Association between PM_2.5_ exposure defined by different time duration and cardiovascular risk factors prevalence.**

The odds ratios and relevant 95% CI were scaled to each 10μg/m^3^ PM_2.5_ exposure and calculated by multivariable logistic regression, and further adjusted for age, sex, education, ethnicity, smoking status, drinking status, intensity of physical activity, and diet types.

**Figure S8 Association between dichotomous PM_2.5_ exposure and cardiovascular risk factors prevalence.**

PM_2.5_ were categorized into “high” or “low” by a threshold of 35 μg/m^3^. The low exposure group was used as the reference group. The odds ratios and relevant 95% CI were scaled to dichotomous PM_2.5_ exposure and calculated by multivariable logistic regression, and further adjusted for age, sex, education, ethnicity, smoking status, drinking status, intensity of physical activity, and diet types.

**Figure S9 Association between PM2.5 exposure and cardiovascular risk factors prevalence stratified by residence (urban or rural).**

The odds ratios and relevant 95% CI were scaled to each 10μg/m^3^ PM_2.5_ exposure and calculated by multivariable logistic regression, and further adjusted for age, sex, education, ethnicity, smoking status, drinking status, intensity of physical activity, and diet types.

**Figure S10 Association between PM_2.5_ exposure and cardiovascular risk factors prevalence stratified by region (south or north).**

The odds ratios and relevant 95% CI were scaled to each 10μg/m^3^ PM_2.5_ exposure and calculated by multivariable logistic regression, and further adjusted for age, sex, education, ethnicity, smoking status, drinking status, intensity of physical activity, and diet types.

**Figure S11 Association between PM_2.5_ exposure and cardiovascular risk factors prevalence**

The odds ratios and relevant 95% CI were scaled to each 10μg/m^3^ PM_2.5_ exposure and calculated by multivariable logistic regression in crude model, and further adjusted for age, sex, education, ethnicity, residence (urban or rural), smoking status, drinking status, intensity of physical activity, and diet types in adjusted model.

**Figure S12 Association between PM_2.5_ exposure and cardiovascular risk factors prevalence stratified by covariates.**

Population was stratified by age, sex, education, ethnicity, smoking status, drinking status, intensity of physical activity, and diet types. The odds ratios and relevant 95% CI were scaled to each 10μg/m^3^ PM_2.5_ exposure and calculated by multivariable logistic regression, and further adjusted for age, sex, education, ethnicity, residence (rural or urban), smoking status, drinking status, intensity of physical activity, and diet types. The significance of interaction effect was tested by introducing an interaction term in the regression model.

**Figure S13 Exposure-response relationship between PM_2.5_ exposure and cardiovascular risk factors prevalence in population with preliminary or below education.**

(A) diabetes; (B) hypertension; (C) hyperlipidemia; (D) being overweight. The exposure-response relationship was calculated by generalized additive model, and further adjusted by age, sex, ethnicity, residence (rural or urban), smoking status, drinking status, intensity of physical activity, diet types. Knots used in the generalized additive model was 3. P value was denoted in each panel.

**Figure S14 Association between PM_2.5_ exposure and cardiovascular risk factors prevalence stratified by cardiovascular risk factors.**

The odds ratios and relevant 95% CI were scaled to each 10μg/m^3^ PM_2.5_ exposure and calculated by multivariable logistic regression, and further adjusted for age, sex, education, ethnicity, residence (urban or rural), smoking status, drinking status, intensity of physical activity, and diet types.

# Table S1 Prevalence of cardiovascular risk factors in urban and rural residents

| Disease | **Overall population** | **Urban** | **Rural** | *P* |
| --- | --- | --- | --- | --- |
| N, *n* (%) | 19,236 (100) | 9,777 (50.8) | 9,459 (49.2) |  |
| Diabetes, *n* (%) *^a^* | 755 (3.9) | 369 (3.8) | 386 (4.1) | 0.290 |
| Hypertension, *n* (%) *^a^* | 4,383 (22.8) | 1,964 (20.1) | 2,419 (25.6) | <0.001 |
| Hyperlipidemia, *n* (%) *^a^* | 5,391 (28.0) | 2,469 (25.3) | 2,922 (30.9) | <0.001 |
| Overweight, *n* (%) *^a^* | 7,727 (40.2) | 3,898 (39.9) | 3,829 (40.5) | 0.396 |

*^a^* Compared by the χ2 test.

# Table S2 Prevalence of cardiovascular risk factors in north and south region

| Disease | **Overall population** | **South** | **North** | *P* |
| --- | --- | --- | --- | --- |
| N, *n* (%) | 19,236 (100) | 9,459 (49.2) | 9,777 (50.8) |  |
| Diabetes, *n* (%) *^a^* | 755 (3.9) | 390 (3.8) | 365 (4.1) | 0.422 |
| Hypertension, *n* (%) *^a^* | 4,383 (22.8) | 2,235 (21.8) | 2,148 (23.8) | 0.001 |
| Hyperlipidemia, *n* (%) *^a^* | 5,391 (28.0) | 3,015 (29.5) | 2,376 (26.4) | <0.001 |
| Overweight, *n* (%) *^a^* | 7,727 (40.2) | 3,441 (33.7) | 4,286 (47.6) | <0.001 |

*^a^* Compared by the χ2 test.


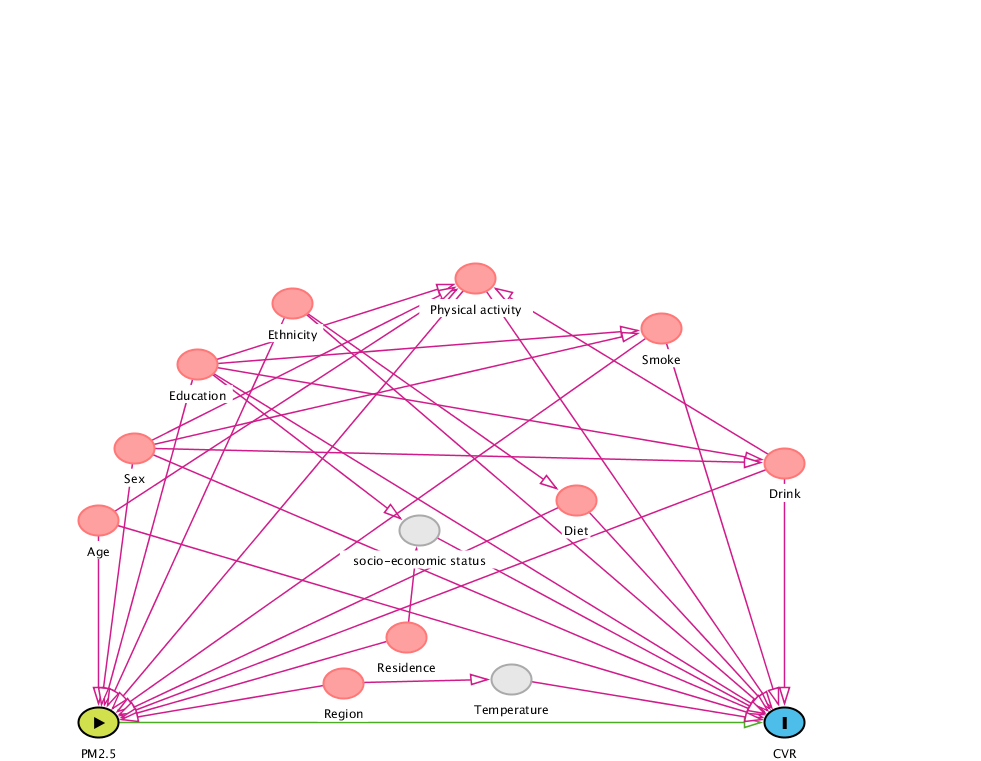


# Figure S1 Proposed Directed acyclic graph for the association between PM_2.5_ and CVR (cardiovascular risk factors)

**
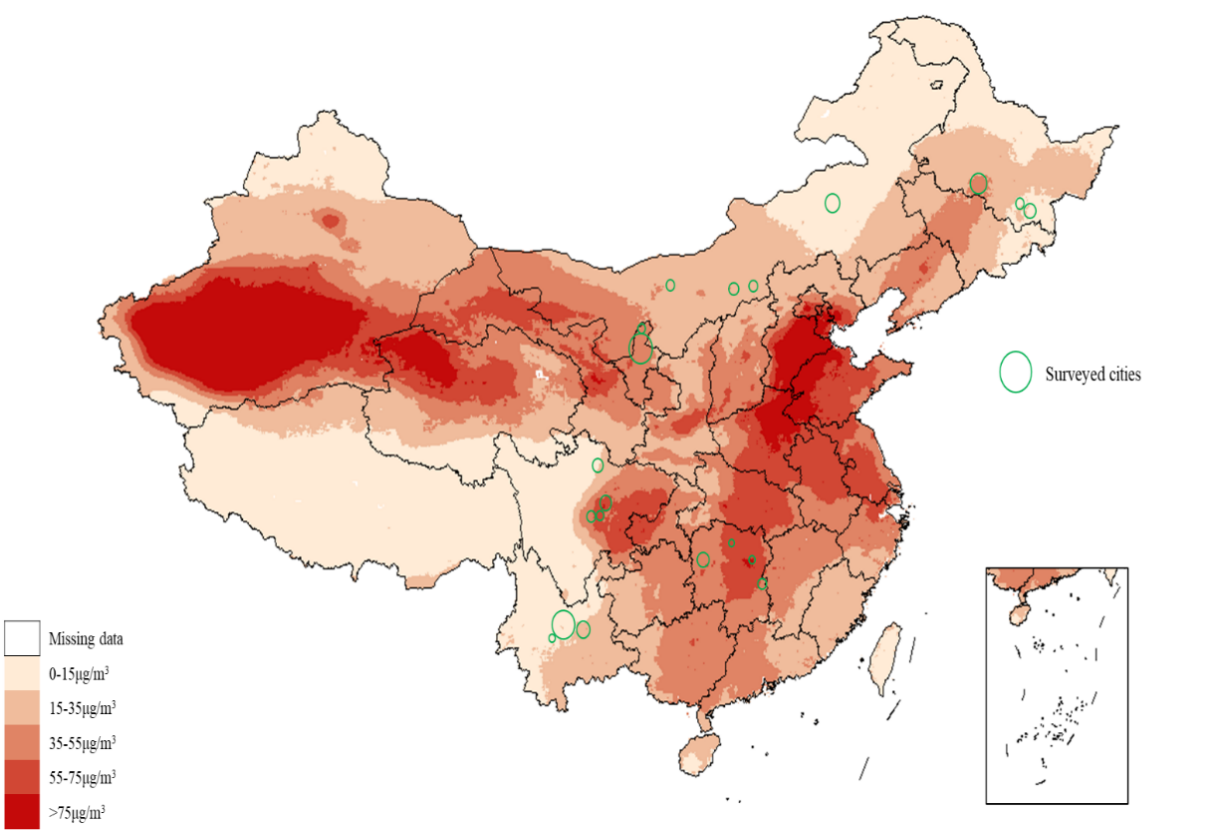
**

# Figure S2 Annual average PM_2.5_ concentration in 2008, China and distribution of surveyed cities.


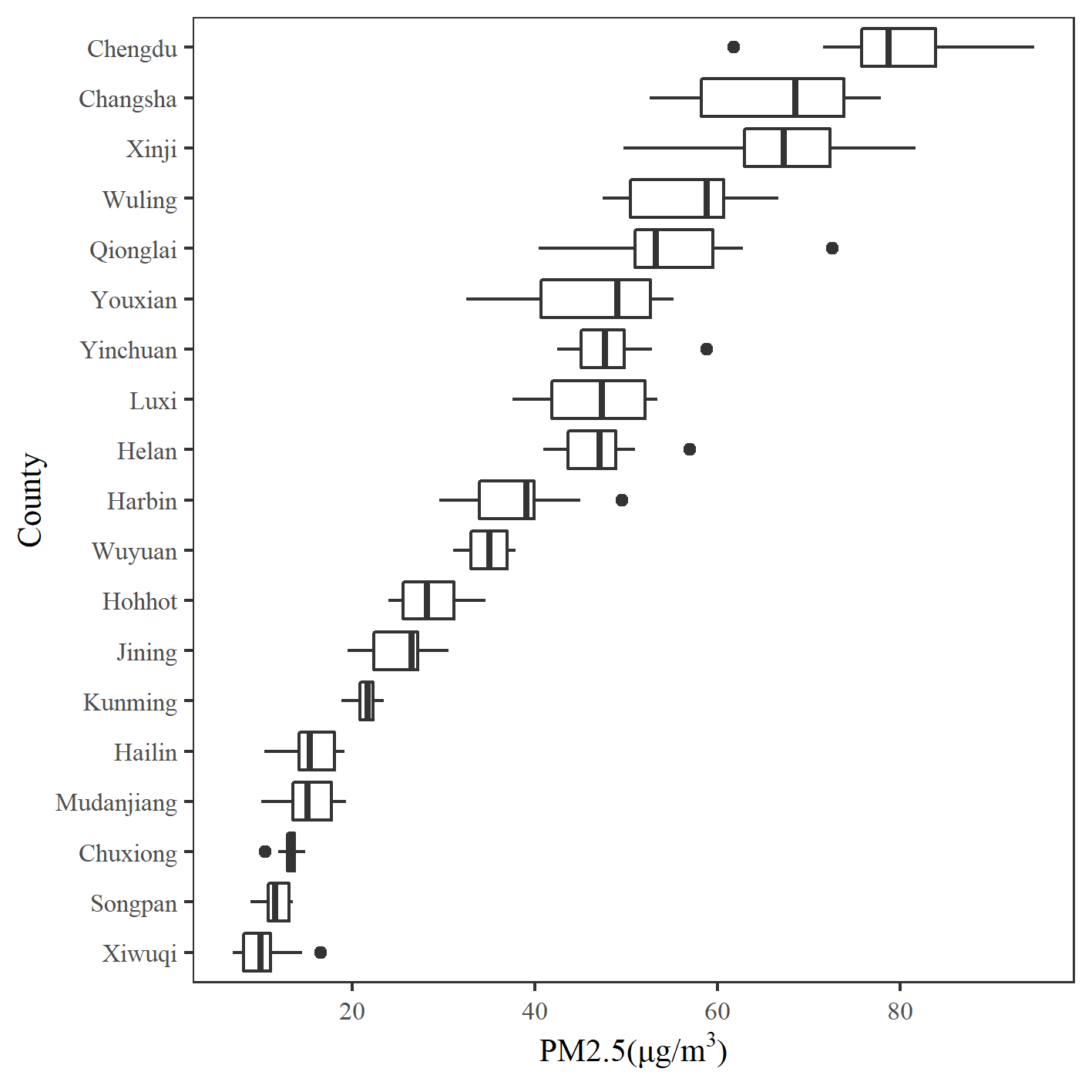


# Figure S3 Distributions of PM_2.5_ concentration in the surveyed cities (2001-2010).

Boxes covered the 25–75th percentile, with a centerline for the median concentration. Whiskers extend to the highest observation within 3 IQRs (interquartile ranges) of each box. The extreme data were shown as dots.


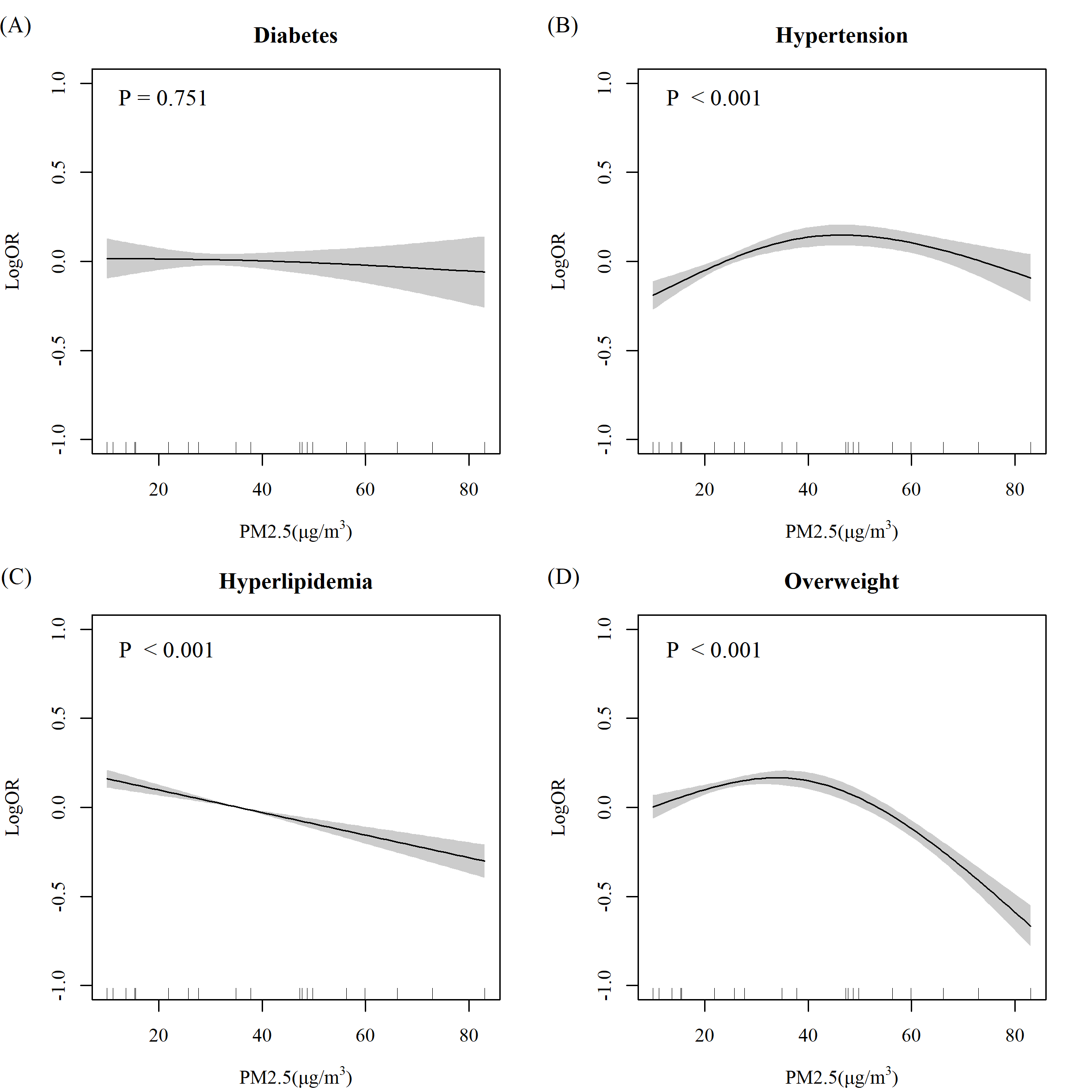


# Figure S4 Exposure-response relationship between PM_2.5_ exposure and cardiovascular risk factors prevalence in general population.

(A) diabetes; (B) hypertension；(C) hyperlipidemia; (D) overweight. The exposure-response relationship was calculated by generalized additive model, and further adjusted by age, sex, education, ethnicity, smoking status, drinking status, intensity of physical activity, diet types. Knots used in the generalized additive model was 3. P value was denoted in each panel.


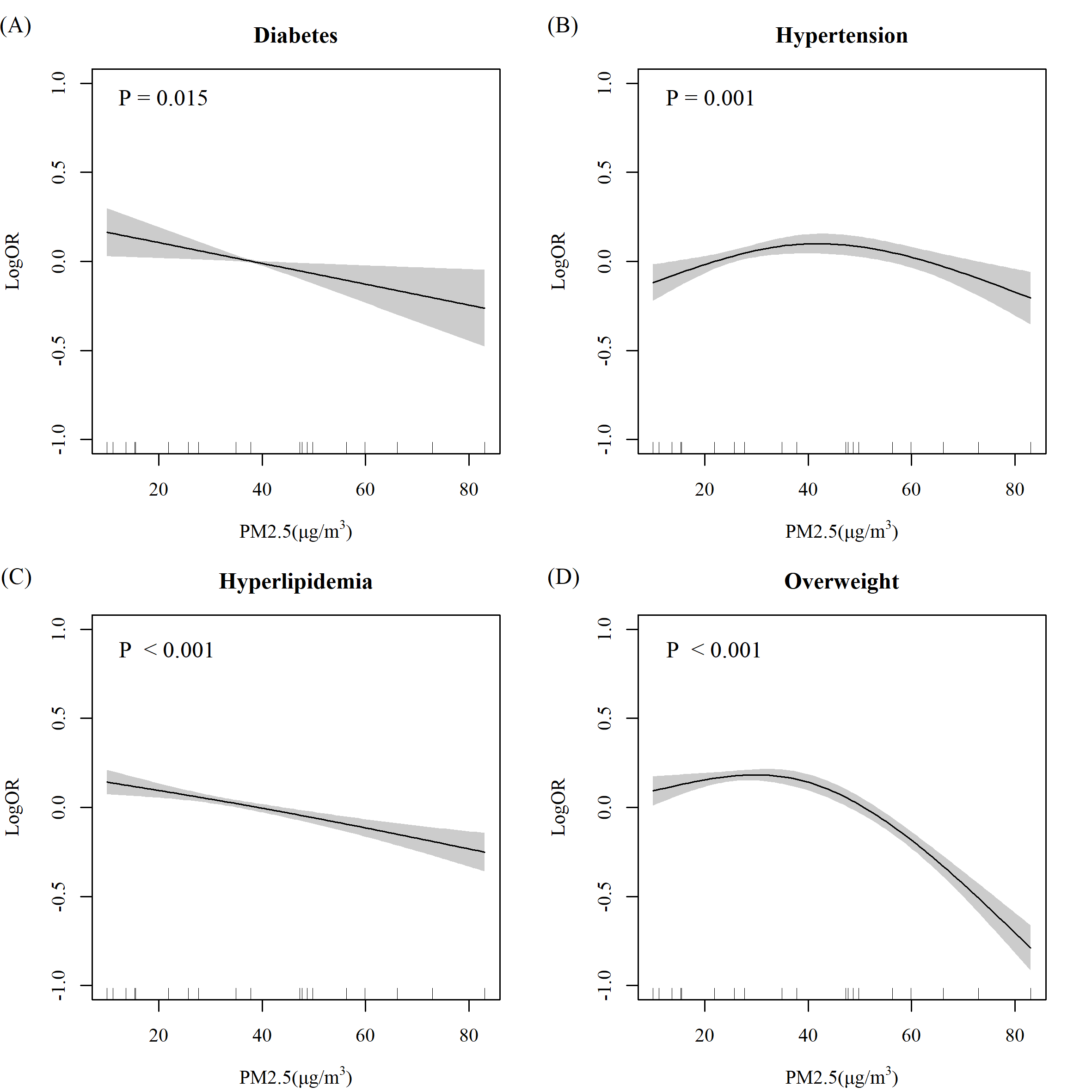


# Figure S5 Exposure-response relationship between PM_2.5_ exposure and cardiovascular risk factors prevalence in population with middle or above education.

(A) diabetes; (B) hypertension; (C) hyperlipidemia; (D) being overweight. The exposure-response relationship was calculated by generalized additive model, and further adjusted by age, sex, ethnicity, smoking status, drinking status, intensity of physical activity, diet types. Knots used in the generalized additive model was 3. P value was denoted in each panel.


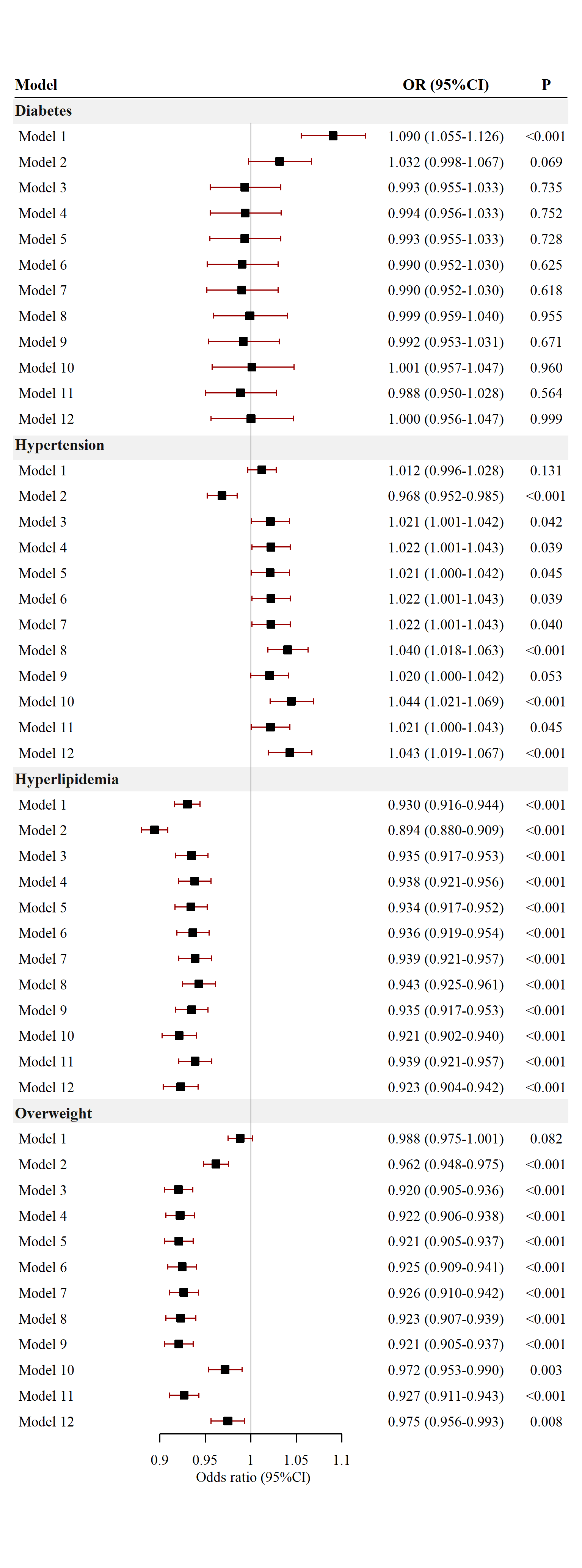


# Figure S6 Association between PM_2.5_ exposure and cardiovascular risk factors prevalence determined by different logistic regression models.

The odds ratios and relevant 95% CI were scaled to each 10μg/m^3^ PM_2.5_ exposure and calculated by univariate or multivariable logistic regression.

Model 1 adjusted for: none

Model 2 adjusted for: age, sex

Model 3 adjusted for: Model 2 + education, ethnicity

Model 4 adjusted for: Model 3 + smoking status, drinking status

Model 5 adjusted for: Model 3 + intensity of physical activity

Model 6 adjusted for: Model 3 + diet types

Model 7 adjusted for: Model 3 + smoking status, drinking status, intensity of physical activity, diet types

Model 8 adjusted for: Model 7 + all other diseases

Model 9 adjusted for: Model 3 + residence (rural/ urban)

Model 10 adjusted for: Model 3 + region (south/ north)

Model 11 adjusted for: Model 7 + residence (rural/ urban)

Model 12 adjusted for: Model 7 + region (south/ north)


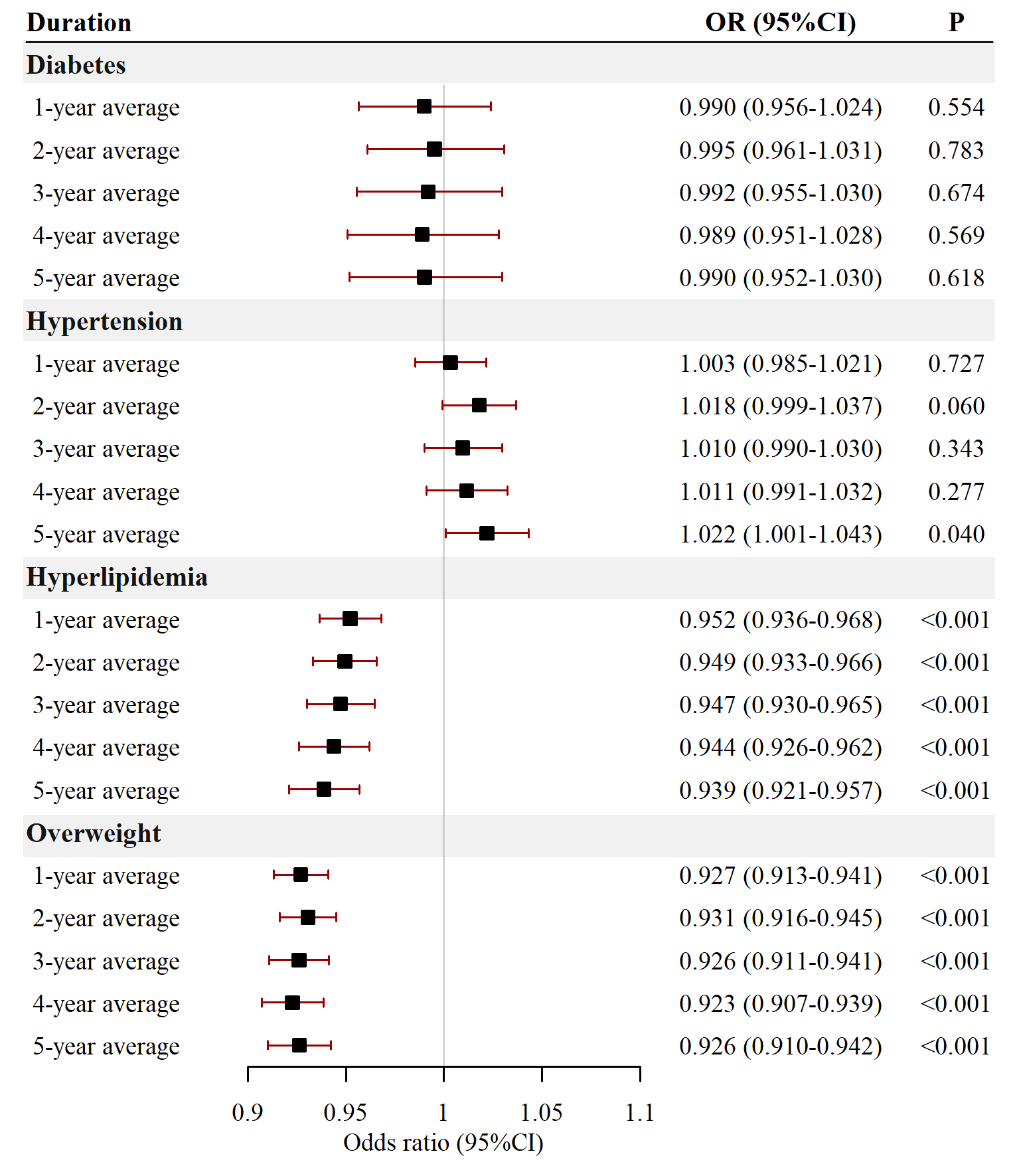


# Figure S7 Association between PM_2.5_ exposure defined by different time duration and cardiovascular risk factors prevalence.

The odds ratios and relevant 95% CI were scaled to each 10μg/m^3^ PM_2.5_ exposure and calculated by multivariable logistic regression, and further adjusted for age, sex, education, ethnicity, smoking status, drinking status, intensity of physical activity, and diet types.


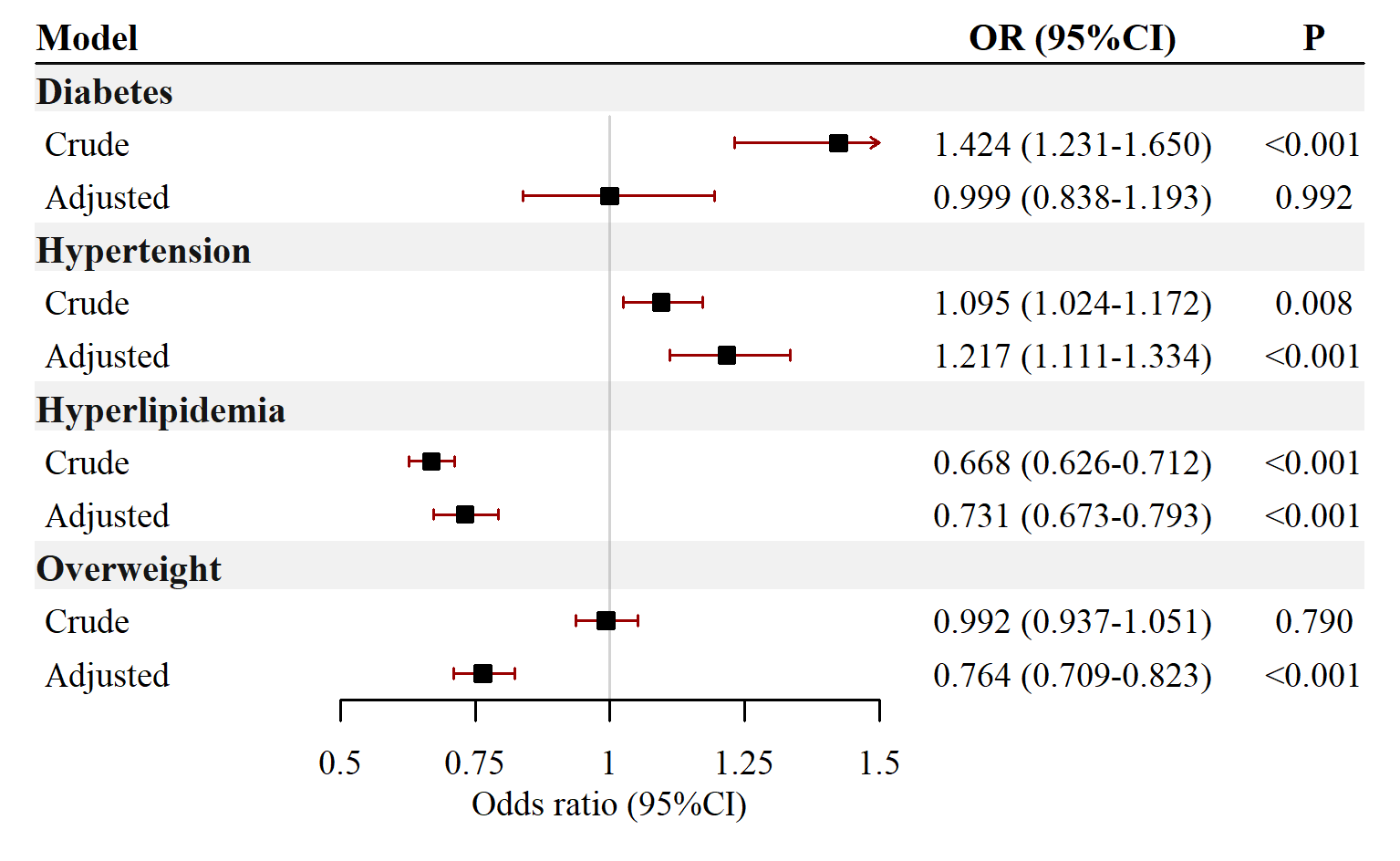


# Figure S8 Association between dichotomous PM_2.5_ exposure and cardiovascular risk factors prevalence.

PM_2.5_ were categorized into “high” or “low” by a threshold of 35 μg/m^3^. The low exposure group was used as the reference group. The odds ratios and relevant 95% CI were scaled to dichotomous PM_2.5_ exposure and calculated by multivariable logistic regression, and further adjusted for age, sex, education, ethnicity, smoking status, drinking status, intensity of physical activity, and diet types.


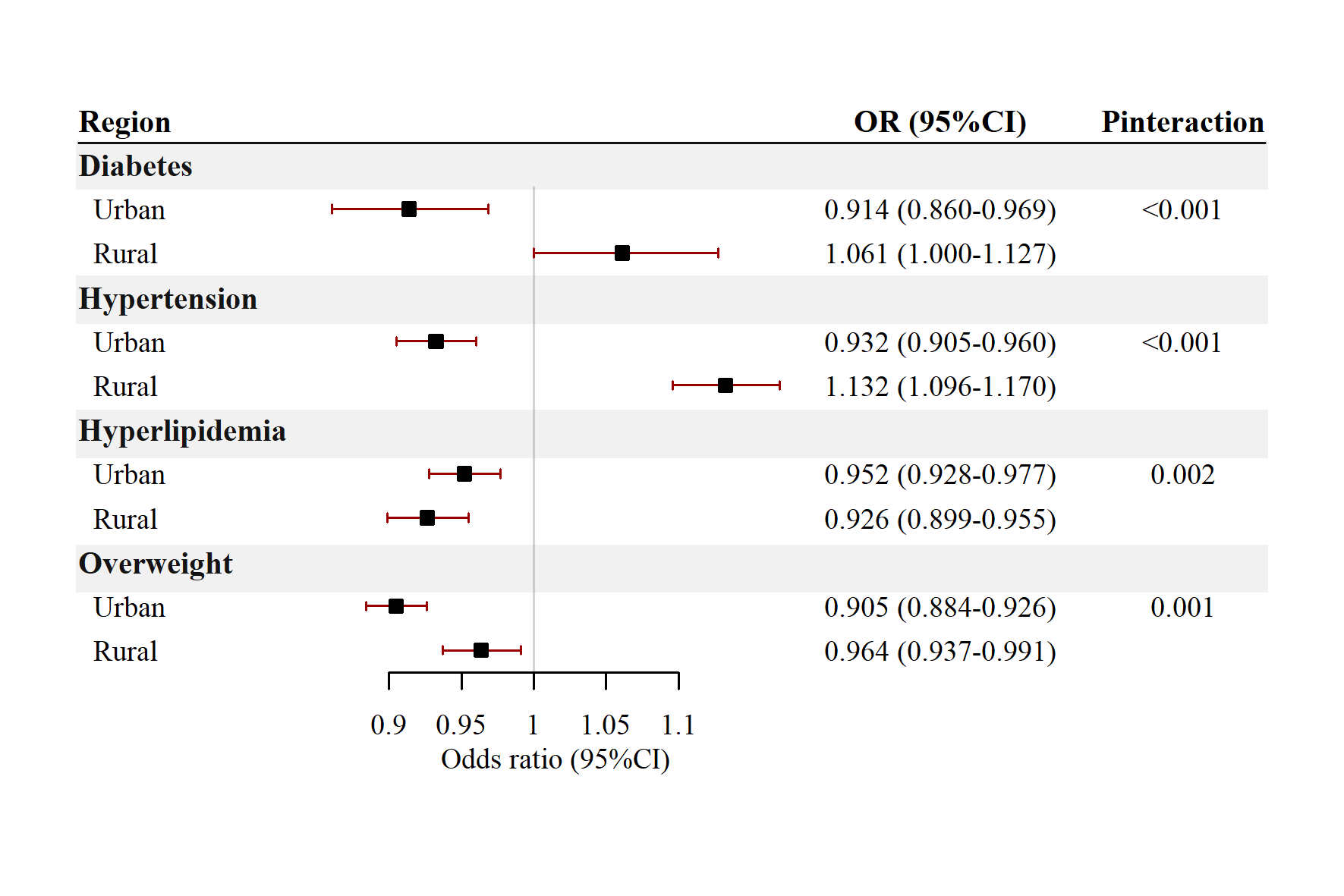


# Figure S9 Association between PM_2.5_ exposure and cardiovascular risk factors prevalence stratified by residence (urban or rural).

The odds ratios and relevant 95% CI were scaled to each 10μg/m^3^ PM_2.5_ exposure and calculated by multivariable logistic regression, and further adjusted for age, sex, education, ethnicity, smoking status, drinking status, intensity of physical activity, and diet types.


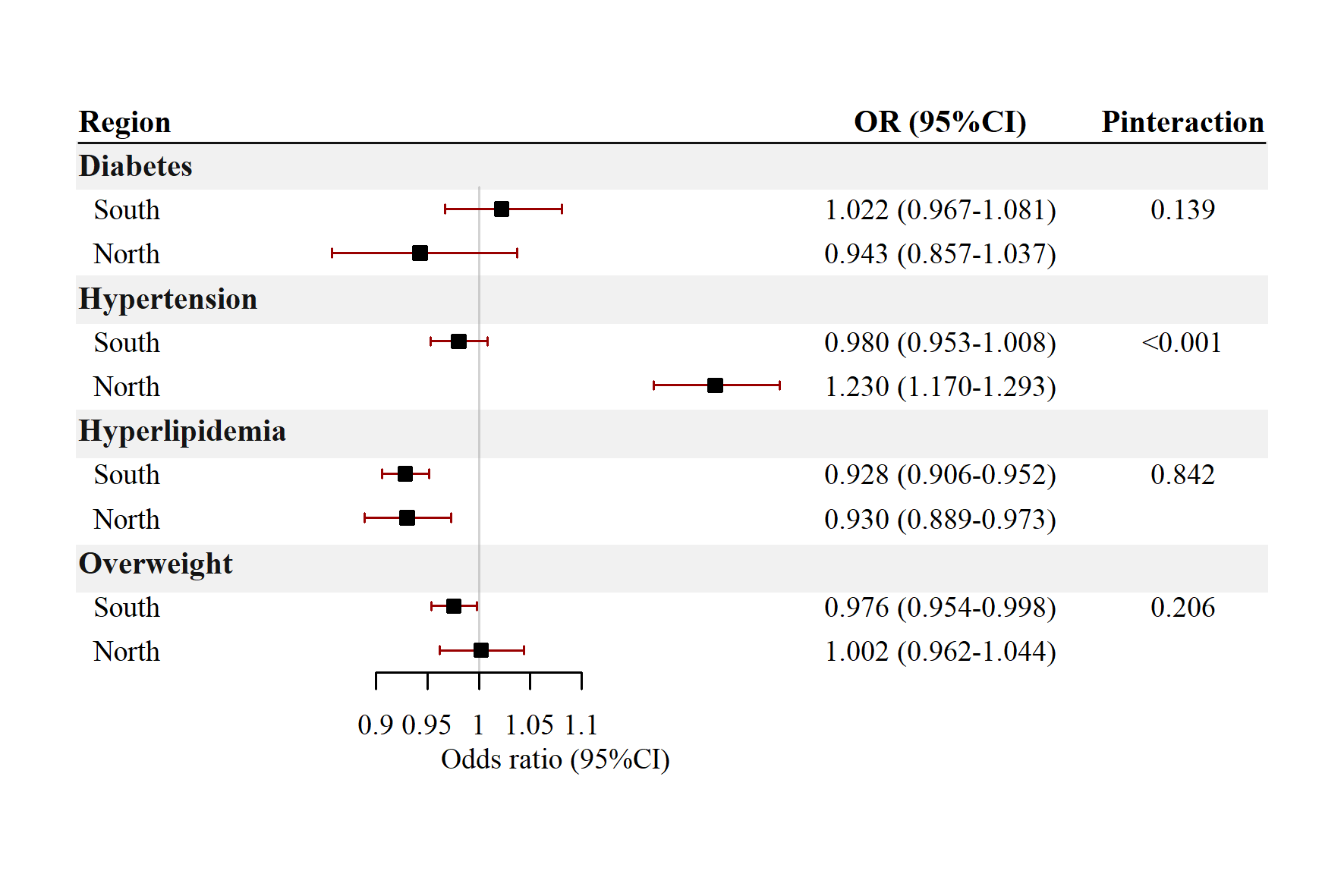


# Figure S10 Association between PM_2.5_ exposure and cardiovascular risk factors prevalence stratified by region (south or north).

The odds ratios and relevant 95% CI were scaled to each 10μg/m^3^ PM_2.5_ exposure and calculated by multivariable logistic regression, and further adjusted for age, sex, education, ethnicity, smoking status, drinking status, intensity of physical activity, and diet types.


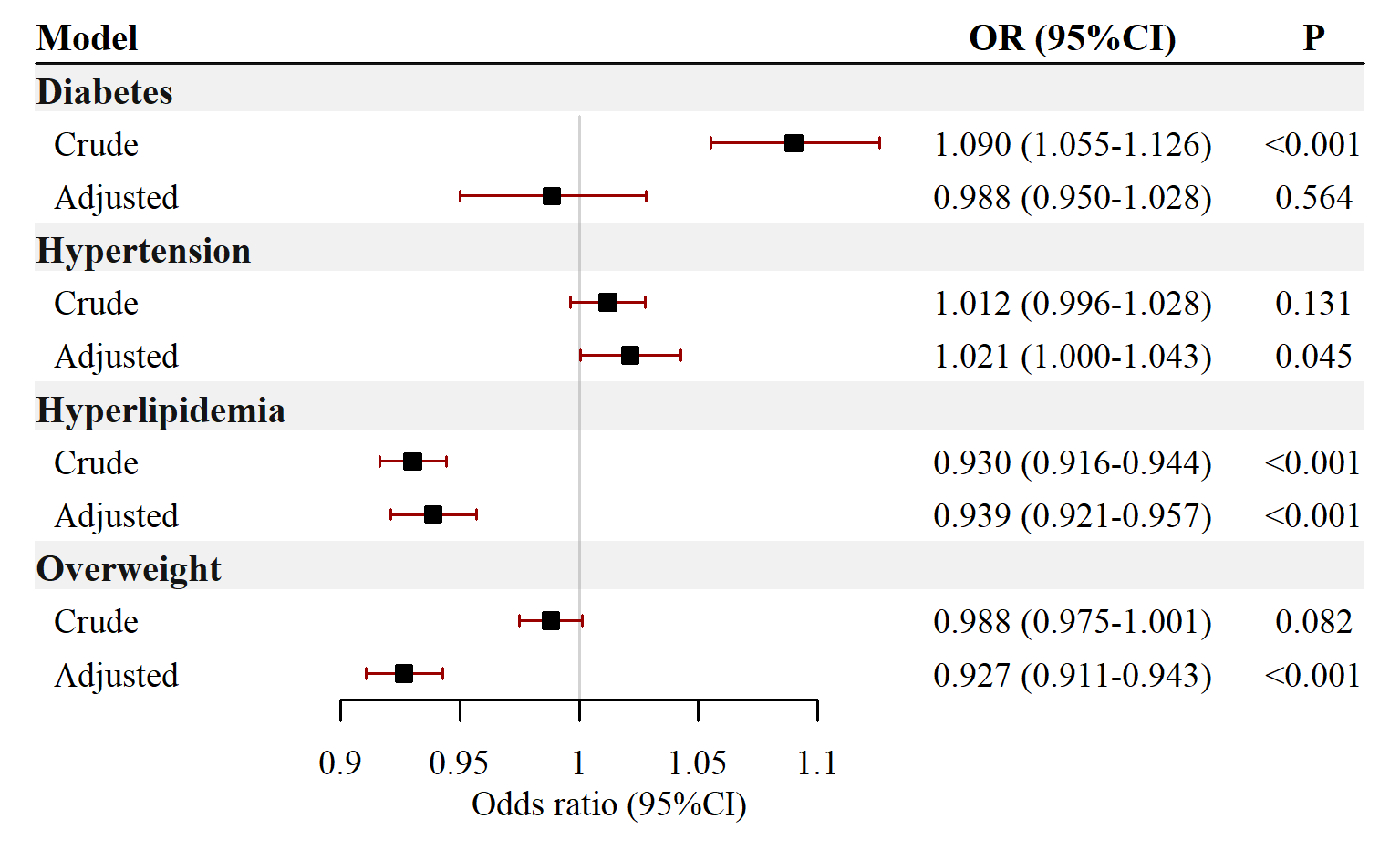


**Figure S11 Association between PM_2.5_ exposure and cardiovascular risk factors prevalence**

The odds ratios and relevant 95% CI were scaled to each 10μg/m^3^ PM_2.5_ exposure and calculated by multivariable logistic regression in crude model, and further adjusted for age, sex, education, ethnicity, residence (urban or rural), smoking status, drinking status, intensity of physical activity, and diet types in adjusted model.


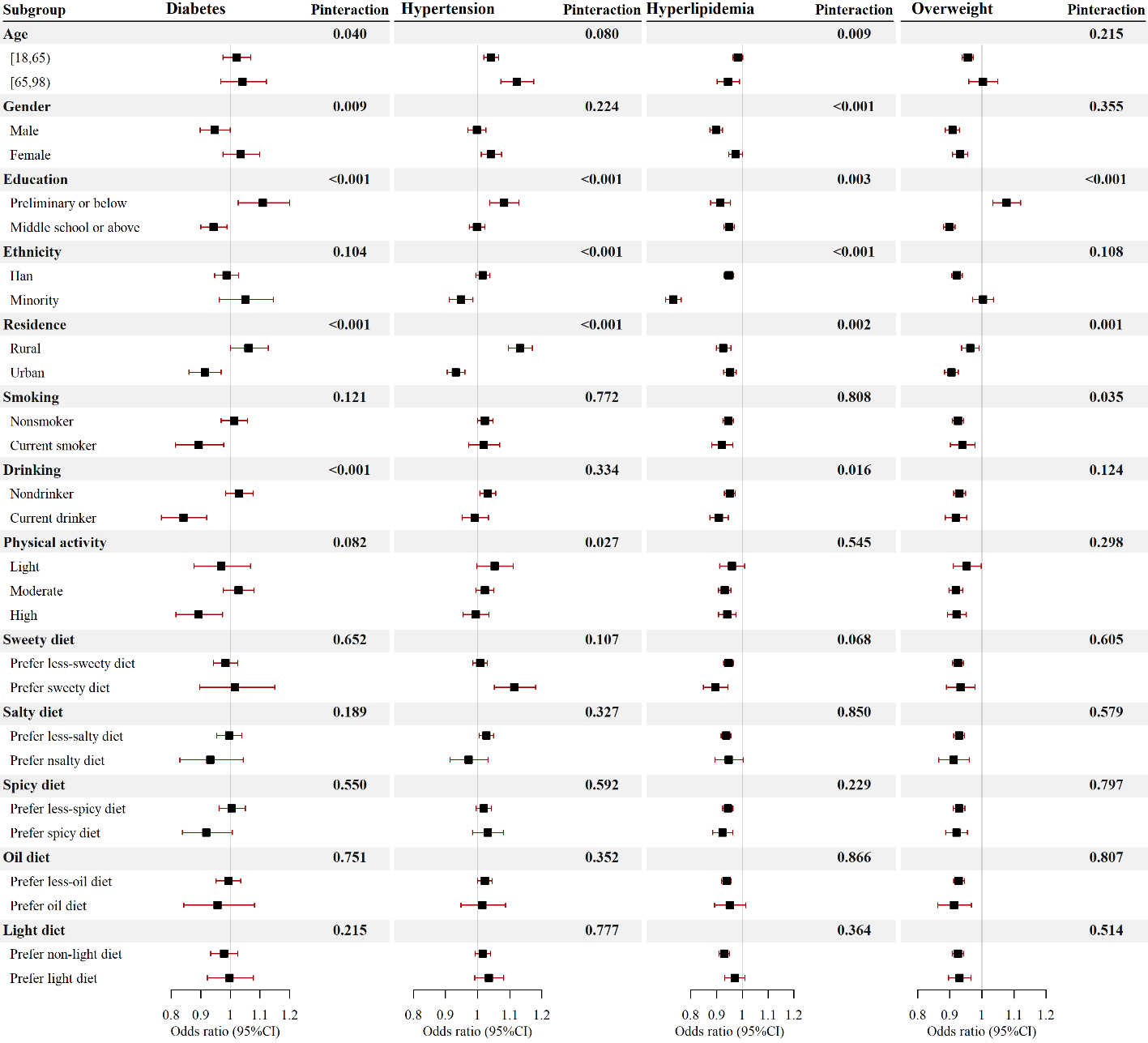


**Figure S12 Association between PM_2.5_ exposure and cardiovascular risk factors prevalence stratified by covariates.**

Population was stratified by age, sex, education, ethnicity, smoking status, drinking status, intensity of physical activity, and diet types. The odds ratios and relevant 95% CI were scaled to each 10μg/m^3^ PM_2.5_ exposure and calculated by multivariable logistic regression, and further adjusted for age, sex, education, ethnicity, residence (rural or urban), smoking status, drinking status, intensity of physical activity, and diet types. The significance of interaction effect was tested by introducing an interaction term in the regression model.


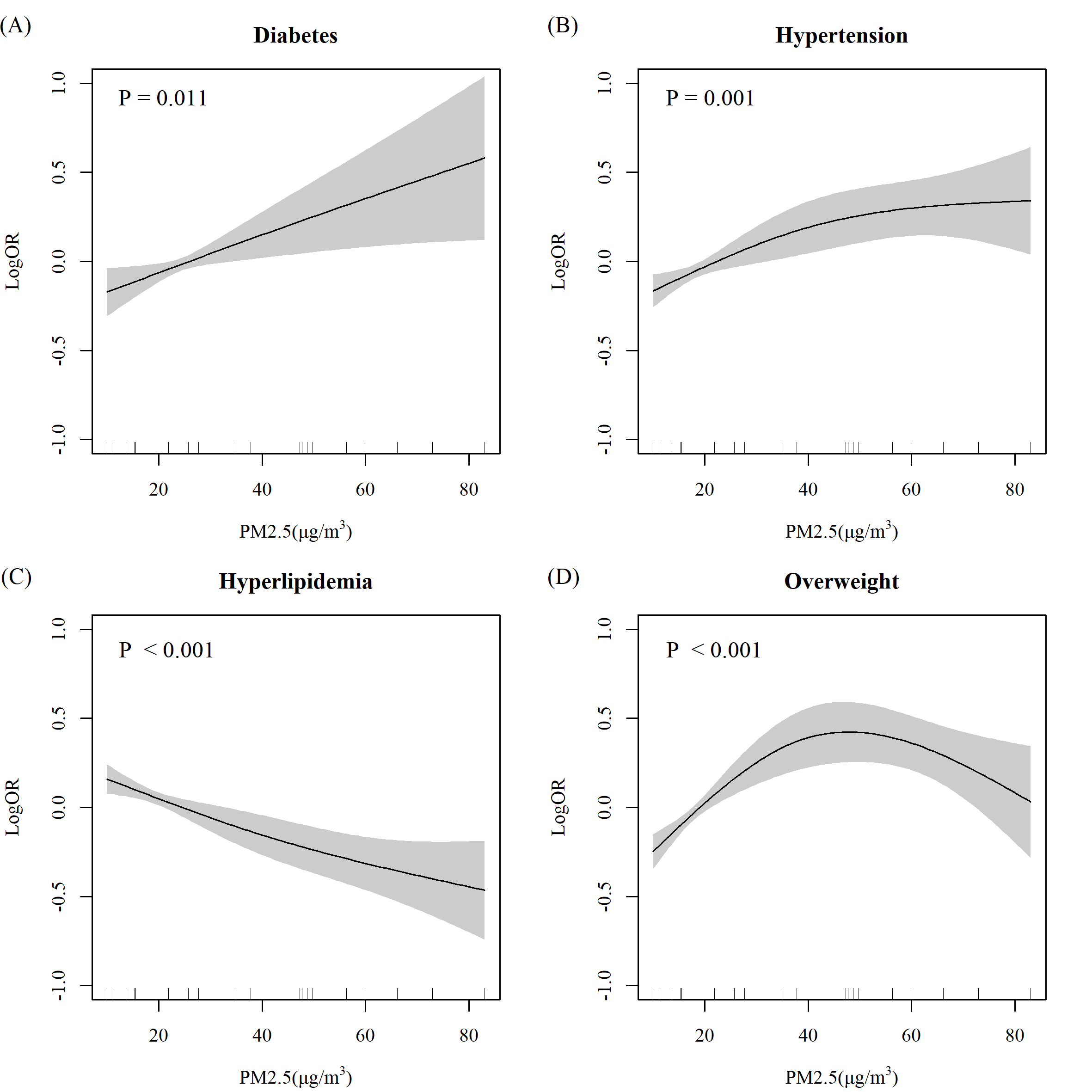


**Figure S13 Exposure-response relationship between PM_2.5_ exposure and cardiovascular risk factors prevalence in population with preliminary or below education.**

(A) diabetes; (B) hypertension; (C) hyperlipidemia; (D) being overweight. The exposure-response relationship was calculated by generalized additive model, and further adjusted by age, sex, ethnicity, residence (rural or urban), smoking status, drinking status, intensity of physical activity, diet types. Knots used in the generalized additive model was 3. P value was denoted in each panel.


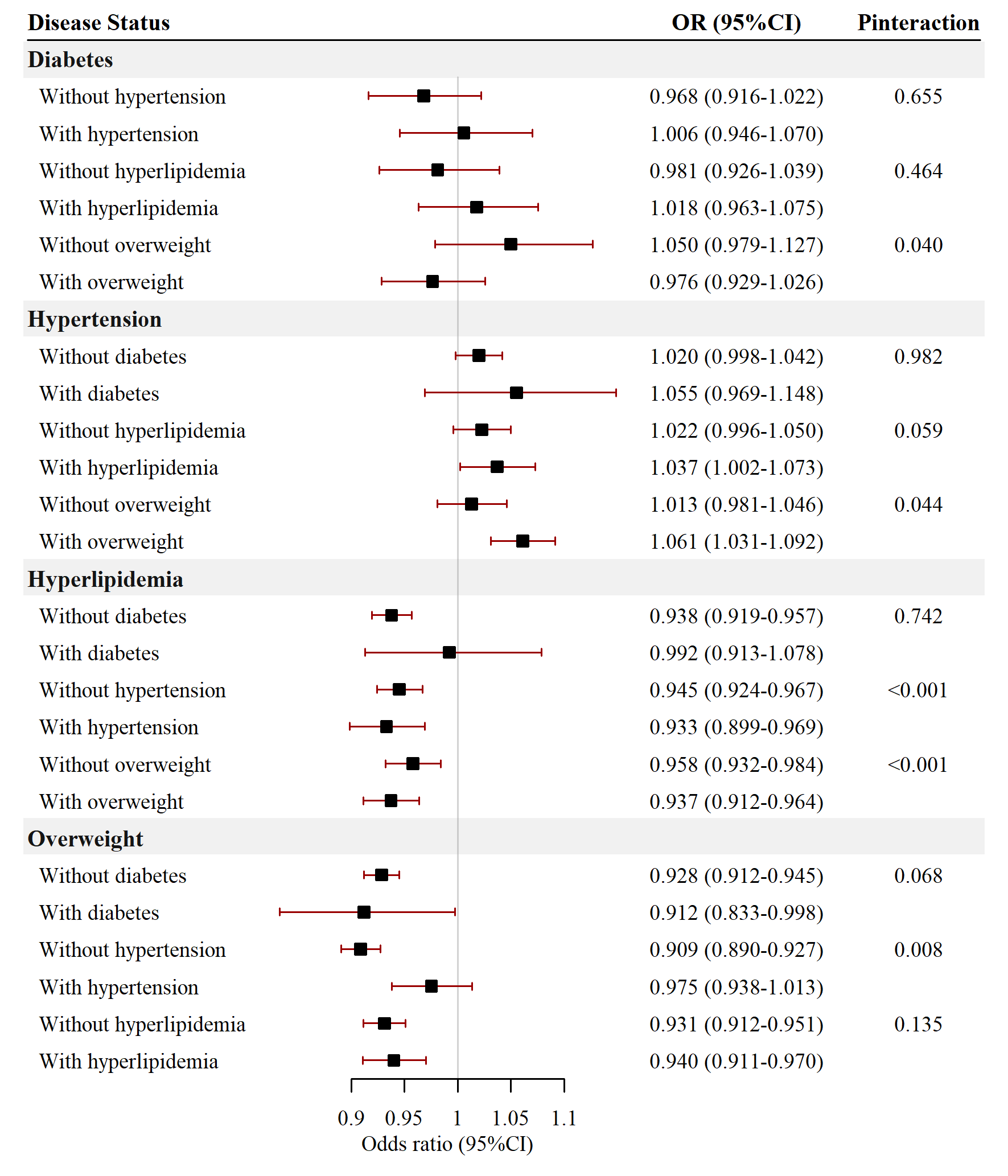


**Figure S14 Association between PM_2.5_ exposure and cardiovascular risk factors prevalence stratified by cardiovascular risk factors.**

The odds ratios and relevant 95% CI were scaled to each 10μg/m^3^ PM_2.5_ exposure and calculated by multivariable logistic regression, and further adjusted for age, sex, education, ethnicity, residence (urban or rural), smoking status, drinking status, intensity of physical activity, and diet types.
